# Letter to the Editor: “A novel concealed posterior axillary approach for scapula glenoid fractures - a multicenter clinical study”

**DOI:** 10.1097/JS9.0000000000004713

**Published:** 2026-01-13

**Authors:** Zhihui Xu, Jingwen Ma, Dapeng Ding

**Affiliations:** a Department of Clinical Laboratory Medicine, The First Affiliated Hospital of Dalian Medical University, Dalian, China; b College of Laboratory Medicine, Dalian Medical University, Dalian, China

*Dear Editor,*

We read with great interest the article published in the *International Journal of Surgery* entitled “A novel concealed posterior axillary approach for scapula glenoid fractures-a multicenter clinical study”[1]. The study’s principal innovation lies in introducing the Tian Yun-Wu Dankai (T-W) approach, a concealed posterior axillary access for the management of scapular glenoid fractures. This technique offers potential clinical advantages, including minimal invasiveness, reduced operative time, stable fixation, favorable functional recovery, and cosmetically hidden scarring. Nevertheless, we have identified several limitations that warrant further consideration. This article was written following the TITAN guidelines[2].

First, the stratified analysis across groups appeared insufficient. The authors categorized patients according to the Ideberg classification and evaluated postoperative functional recovery using the Constant and Disabilities of the Arm, Shoulder and Hand Scale (DASH) scores. However, substantial discrepancies existed among the groups: Ideberg type Ia cases (*n* = 71) involved relatively mild anterior glenoid rim fractures, whereas types IV/V (*n* = 6) affected multiple regions of the glenoid and scapula, representing more complex injuries with differing operative difficulty and surgical trauma[3]. Consequently, calculating overall functional scores may have diluted the true outcomes of the Ideberg IV/V subgroup. In addition, this study has not addressed the applicability of the T-W approach in elderly patients with osteoporosis or individuals with extreme BMI values. Osteoporotic patients are more prone to fixation loosening, while excessively high or low BMI may compromise the surgical field and the effectiveness of muscle separation^[4,5]^. Conducting stratified analyses based on injury severity, age, and BMI could help reduce heterogeneity and further strengthen the validity and interpretability of the findings.

Second, the study lacked both a control group and an assessment of cost implications. Without a comparison cohort using traditional surgical approaches, the relative advantages of the T-W approach cannot be fully established. We suggest that cadaveric studies employing conventional approaches could help evaluate and compare the effects of both techniques on shoulder joint stability, which would provide valuable clinical insight. Furthermore, the study did not include an analysis of economic cost. The T-W approach may require the use of double plates and anchors, potentially increasing material expenses compared with traditional methods. Incorporating a cost comparison would therefore strengthen the comprehensiveness of the study. In addition, the feasibility of the T-W approach in cases of secondary fractures presents further challenges that warrant discussion.

Finally, the recording of intraoperative variables and the multidimensional postoperative assessment appeared insufficient. Important parameters such as the number of capsular incisions, the type of incision used, and the frequency of intraoperative fluoroscopy were not documented, although these factors may influence postoperative outcomes. Moreover, although the T-W approach preserves tendon integrity, the required latissimus dorsi–rotator cuff separation may still affect postoperative shoulder muscle function[6]. Objective evaluations of scapular function and neuromuscular coordination-such as surface electromyography and dynamic ultrasound-would therefore enhance the assessment of functional recovery. In addition, postoperative evaluation in the study relied solely on radiographs and computed tomography (CT) scans to assess fracture reduction. Incorporating shoulder-specific functional scores, such as the American Shoulder and Elbow Surgeon’s Form (ASES) score, would provide a more comprehensive, multidimensional appraisal of postoperative status.

In conclusion, the T-W approach represents an innovative option for the management of scapular glenoid fractures, offering the advantages of minimal invasiveness and stable fixation. Nevertheless, several issues remain, including limited stratified analyses, the absence of a control group and cost evaluation, insufficient documentation of key intraoperative variables, and a lack of multidimensional postoperative functional assessments. Addressing these aspects in future studies would help further substantiate the safety and effectiveness of the T-W approach and enhance its clinical applicability.

## Data Availability

Data sharing is not applicable to this article as no new data were created or analyzed in this study.
